# A cardiac arrest on a spiked helmet electrocardiographic sign in anterior leads in a patient with a diagnosis of acute myocardial infarction: A case report

**DOI:** 10.1016/j.amsu.2022.103635

**Published:** 2022-04-15

**Authors:** Zakariae Belarbi, Falmata Laouan Brem, Noha El Ouafi

**Affiliations:** a Department of Cardiology, Mohammed VI University Hospital, Faculty of Medicine and Pharmacy of Oujda, Mohammed First University, Oujda, MAR, Morocco; b Epidemiological Laboratory of Clinical Research and Public Health, Oujda, Morocco

**Keywords:** Case report, Acute myocardial infarction, ST-Segment elevation, Spiked helmet sign, Electrocardiogram

## Abstract

In patients with critical heart disease, such as acute coronary syndrome, aortic dissection, and other diseases, ST-segment elevation is a relatively common finding on the electrocardiogram (ECG). There are various other well-known signs described in heart diseases, such as negative T waves and q waves of necrosis. The “spiked helmet sign” is a novel electrocardiographic sign described first in 2011, whose pathophysiology and clinical applicability remain uncertain at this time. Herein we report the case of a cardiac arrest in a patient who developed the “spiked helmet electrocardiographic sign” concomitantly with acute myocardial infarction, leading to the patient's death from ventricular fibrillation. This case report aims to discuss the “spiked helmet electrocardiographic sign”, and to detail the prognostic and diagnostic interest of this sign, wich seems to be different from a standard ST segment elevation.

## Highlights

• Cardiovascular diseases are the world's first and foremost cause of death.
• The “spiked helmet sign” is a rare electrocardiographic sign recently described in 2011 that can be found in a variety of serious diseases, the most common is the acute coronary syndrome.
• This sign is described as a potential novel ECG marker of a very high risk of impending death.
• This sign may be used in the future to detect critically ill patients, in order to speed up treatment strategies.
• Clinicians must be vigilant in front of such a sign.

## 1 Introduction

Cardiovascular diseases are the world's first and foremost cause of death. There are various tools available for diagnosing these diseases. However, the ECG remains a popular option. The “spiked helmet sign” is an electrocardiographic sign recently described in 2011 that can be found in a variety of serious diseases, the most common is the acute coronary syndrome [1]. The majority of studies that have described this electrical sign have reported a poor overall prognosis [2].

This sign is characterized by a ‘spike and dome’ with elevation in the ECG baseline prior to the R wave and adjoining ST-segment elevation predominantly in the inferior leads (II, III, and aVF) [^1^]. This ECG pattern had a morphology that approximated the shape of a vintage German military spiky helmet employed by the Prussian imperial army in the 19th century. The exact mechanism of the spiked helmet ECG pattern and its link to critical disease is uncertain. Nevertheless, multiple observations indicate the diaphragm as a probable contributor. Others relay the phenomenon to the pulsatile epidermal stretch associated with either acute thoracic or abdominal distension [3].

## 2 Case presentation

A 73-year-old women with a past medical history of hypertension and a recent acute ischemic stroke presented to our center's emergency department (ED) for acute chest pain that began 6 hours before admission. The patient had a normal psychological state, and no history of taking drugs or any other suspicious substance. On arrival, the patient's initial vital signs include a blood pressure of 120/92 mmHg, heart rate of 98 beats per minute, a temperature of 37.9 °C, and a respiratory rate of 15 breaths per minute (oxygen saturation at 98% in room air). On physical examination, the patient appears distressed and slightly diaphoretic. However, the lungs were clear to auscultation bilaterally. Heart sounds revealed a normal S1/S2.

**Fig. 1:**
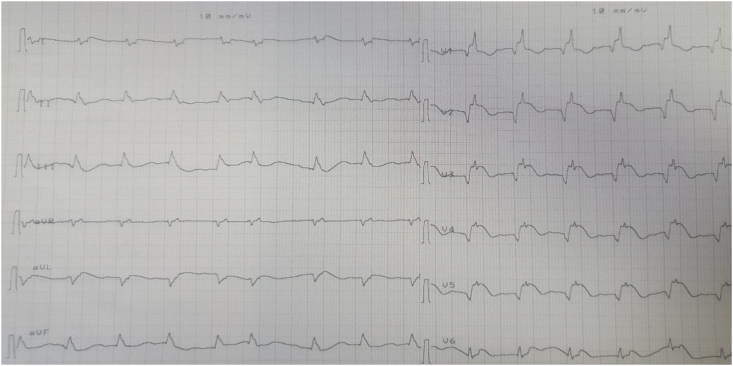
The 12-lead ECG recorded in the critical care unit showing the “spiked helmet” sign.

An initial ECG was performed which shows ST-segment elevation with an upward shift preceding the QRS complexes in the anterior leads V1 V2 V3 V4 V5 [Fig. 1]. Besides, we can see a rhythm on the telemetry monitor consisting of a continuous ST-segment elevation pattern associated with an upsloping preceding the QRS complexes. Dual antiplatelet therapy (Clopidogrel 600mg, Aspirin 300mg) was administered along with 7000 UI of low molecular weight heparin. But unfortunately, the patient rapidly developed ventricular fibrillation 20 min later, followed by unrecovered cardiac arrest after three external electrical shocks and 45 minutes of cardiopulmonary resuscitation. Laboratory results retrieved in post-mortem showed elevated troponin 50 000 ng/l (for a normal value at 27ng/l) and a high D-Dimer level (8.99 ng FEU/L).

## 3 Discussion

Cardiovascular disease is the leading cause of death in the world. There are several indices that we could use to detect these pathologies especially electrocardiographic signs, and some signs are linked to a critical prognosis. To the best of our knowledge, the “spiked helmet sign” is a rare ECG phenomenon. It has been reported in either the peripheral leads or in the inferior leads [1], and more rarely in anterior leads [4]. The spiked helmet sign's specific mechanisms are unknown. It has been documented in patients with significant pathologic conditions marked by a rise in intra-abdominal or intrathoracic pressure [^3^], as well as those with acute cerebrovascular events such as cerebral hemorrhage and subarachnoid hemorrhage [5]. The pulsatile epidermal stretch accompanied with either acute thoracic or abdominal distension is the likely mechanism. Human heart tissue has strain-activated ion channels, which change conductivity under different stretch conditions. Other observations indicate the diaphragm as a probable contributor. Occasionally, certain pathological disorders can cause repetitive diaphragm contraction that is in concert with the cardiac cycle [6,7]. Various acute illnesses are linked to particular localizations of the spiked helmet sign in the 12-lead ECG, such as acute coronary syndrome [8], aortic dissection [9], and subarachnoid hemorrhage [^5^].

In our case, we present a patient who developed the spiked helmet sign in the anterior leads, concomitantly with an acute coronary syndrome, wich finally leads to the patient's death. We already have different electrical signs for such pathologies especially the ST-segment elevation. However clinical practice needs predictive signs of poor prognosis to better distinguish patients at high risk of death. In acute coronary syndrome, for example, current recommendations have insisted on certain delays for initiating revascularization depending on the patient's prognosis, based on criteria and scores that are not always specific to poor prognosis [10]. Therefore, we can see that we do not always have sensitive determinants to decide whether the situation is really severe. According to the literature, the spiked helmet sign may be one of those signs that could be used in the future to detect critically Ill patients. Indeed, in several case report describing the spiked helmet sign, mortality was the fatal outcome for all patients [^2^,^3^,^4^]. According to a case series of 8 patients [^1^], mortality was observed in 6/8 patients 1–10 days after the index ECG, corresponding to a mortality of 75%. Only 2 patients were discharged from hospital, both debilitated, one to a rehabilitation center and one to a skilled nursing facility. Hence all the interest of knowing this diagnostic and prognostic electrical sign, in order to speed up treatment strategies as much as possible and keep a closer follow-up of the patient. Though the clinical applicability of this marker still remain uncertain at this time, and further observations are required.

## 4 Conclusion

This rare case highlights the necessity to be vigilant in front of such an electrical aspect and to remain cautious given the high risk of acute decompensation. Indeed, we can notice that it is associated with many serious pathologies such as acute myocardial infarction, aortic dissection and other severe cardiac and extracardiac pathologies, with a worse prognosis than usual.

## Ethical approval

The patient's family have consented for the publication of this work.

The ethics committee is the deanship of the faculty of medicine mohammed 6 oujda.

## Sources of funding for your research

I didn't receive any funding for this work.

## Author contribution

Z. Belarbi, **CONCEPTION, LITERATURE REVIEW**, **ANALYSIS, DATA COLLECTION, WRITING- REVIEW & EDITING**.

F. Laouan Brem: **CONCEPTION, LITERATURE REVIEW**, **ANALYSIS, DATA COLLECTION, WRITING- REVIEW & EDITING**.

N. El Ouafi: **CONCEPTION, METHODOLOGY, SUPERVISION**.

## Registration of research studies

Name of the registry:
Unique Identifying number or registration ID:
Hyperlink to your specific registration (must be publicly accessible and will be checked):

## Consent

Written informed consent was obtained from the patient for publication of this case report and accompanying images. A copy of the written consent is available for review by the Editor-in-Chief of this journal on request.

## Guarantor

Zakariae Belarbi (corresponding author).

## Provenance and peer review

Not commissioned, externally peer-reviewed.

## Declaration of competing interest

The authors declare non-conflicts of interest.
